# Cyclic Enterobacterial Common Antigen Maintains the Outer Membrane Permeability Barrier of Escherichia coli in a Manner Controlled by YhdP

**DOI:** 10.1128/mBio.01321-18

**Published:** 2018-08-07

**Authors:** Angela M. Mitchell, Tharan Srikumar, Thomas J. Silhavy

**Affiliations:** a Department of Molecular Biology, Princeton University, Princeton, New Jersey, USA; Washington University School of Medicine

**Keywords:** ECA, *Enterobacteriaceae*, *Escherichia coli*, YhdP, enterobacterial common antigen, outer membrane

## Abstract

Gram-negative bacteria have an outer membrane (OM) impermeable to many toxic compounds that can be further strengthened during stress. In *Enterobacteriaceae*, the envelope contains enterobacterial common antigen (ECA), a carbohydrate-derived moiety conserved throughout *Enterobacteriaceae*, the function of which is poorly understood. Previously, we identified several genes in Escherichia coli K-12 responsible for an RpoS-dependent decrease in envelope permeability during carbon-limited stationary phase. For one of these, *yhdP*, a gene of unknown function, deletion causes high levels of both vancomycin and detergent sensitivity, independent of growth phase. We isolated spontaneous suppressor mutants of *yhdP* with loss-of-function mutations in the ECA biosynthesis operon. ECA biosynthesis gene deletions suppressed envelope permeability from *yhdP* deletion independently of envelope stress responses and interactions with other biosynthesis pathways, demonstrating suppression is caused directly by removing ECA. Furthermore, *yhdP* deletion changed cellular ECA levels and *yhdP* was found to co-occur phylogenetically with the ECA biosynthesis operon. Cells make three forms of ECA: ECA lipopolysaccharide (LPS), an ECA chain linked to LPS core; ECA phosphatidylglycerol, a surface-exposed ECA chain linked to phosphatidylglycerol; and cyclic ECA, a cyclized soluble ECA molecule found in the periplasm. We determined that the suppression of envelope permeability with *yhdP* deletion is caused specifically by the loss of cyclic ECA, despite lowered levels of this molecule found with *yhdP* deletion. Furthermore, removing cyclic ECA from wild-type cells also caused changes to OM permeability. Our data demonstrate cyclic ECA acts to maintain the OM permeability barrier in a manner controlled by YhdP.

## IMPORTANCE

Enterobacterial common antigen (ECA) is a surface antigen made by all members of *Enterobacteriaceae*, including many clinically relevant genera (e.g., *Escherichia*, *Klebsiella*, *Yersinia*). Although this surface-exposed molecule is conserved throughout *Enterobacteriaceae*, very few functions have been ascribed to it. Here, we have determined that the periplasmic form of ECA, cyclic ECA, plays a role in maintaining the outer membrane permeability barrier. This activity is controlled by a protein of unknown function, YhdP, and deletion of *yhdP* damages the OM permeability barrier in a cyclic ECA-dependent manner, allowing harmful molecules such as antibiotics into the cell. This role in maintenance of the envelope permeability barrier is the first time a phenotype has been described for cyclic ECA. As the Gram-negative envelope is generally impermeable to antibiotics, understanding the mechanisms through which the barrier is maintained and antibiotics are excluded may lead to improved antibiotic delivery.

## INTRODUCTION

The cellular envelope of Gram-negative bacteria consists of an inner membrane (IM) surrounding the cytoplasm, an asymmetrical outer membrane (OM), and a thin layer of peptidoglycan found in the periplasm separating the two membranes (1). While the inner leaflet of the OM is composed of phospholipids, the outer leaflet is mainly composed of lipopolysaccharide (LPS). LPS possesses a number of negatively charged residues that are bridged by divalent cations to form a strong network of interactions between neighboring LPS molecules (2). Due to these interactions and the amphiphilic nature of LPS, the OM provides the cell a robust permeability barrier, resistant to both large and hydrophobic molecules (3). For this reason, the OM has proven an impediment for the design of new antibiotics to treat Gram-negative bacterial infections.

Enterobacterial common antigen (ECA) is an invariant carbohydrate-derived molecule that is present in the OM and periplasm of members of *Enterobacteriaceae* (4). Although ECA is restricted to one family of bacteria, four of the seven species identified by the World Health Organization as being of high concern due to frequent antibiotic-resistant infections are members of this family (Klebsiella pneumoniae, Escherichia coli, nontyphoidal *Salmonella*, and *Shigella* species) (5). Despite the conserved nature of this molecule within *Enterobacteriaceae* (6), its function is largely unknown. In part, this is because the biosynthesis pathways for ECA, O antigen, and peptidoglycan overlap in such a way that gene deletions preventing ECA biosynthesis often also prevent O-antigen production (7–9) or perturb peptidoglycan biosynthesis, causing envelope stress responses to be activated (i.e., Cpx, Rcs, σ^E^) (10–12). Thus, in interpreting the results of high-throughput screens (13–16), it is difficult to determine whether phenotypes are directly related to the presence or absence of ECA or are instead related to changes to other aspects of the cell envelope. Nevertheless, it is thought that ECA plays a small role in bile salt resistance and in organic acid resistance (17, 18). It is generally assumed that the surface-exposed forms of ECA are responsible for these phenotypes. In addition, in Salmonella enterica serovar Typhimurium, O-antigen and ECA biosynthesis are not genetically connected, and the first gene in ECA biosynthesis, *wecA*, can be deleted without activating stress responses, affecting O-antigen biosynthesis, or impairing peptidoglycan biosynthesis (19). Studies in this strain have demonstrated that cells without ECA are deficient in pathogenesis (19), suggesting that ECA plays an important role in the host.

The structure of ECA is conserved throughout *Enterobacteriaceae*, with each unit of ECA consisting of GlcNAc (*N*-acetylglucosamine), ManNAcA (*N*-acetyl-D-mannosaminuronic acid), and Fuc4NAc (4-acetamido-4,6-dideoxy-d-galactose) (20, 21). The pathway of ECA biosynthesis is analogous to that of O-antigen biosynthesis (see Fig. S1 in the supplemental material). GlcNAc-1-phosphate is linked to undecaprenyl-phosphate (Und-P), a lipid carrier in the IM also used for the biosynthesis of O antigen, peptidoglycan, and capsule carbohydrates, and then ManNAcA and Fuc4NAc are attached (22, 23). Many genes in the ECA biosynthesis operon are responsible for synthesizing these sugars and linking them to Und-P (22, 24, 25). The ECA unit linked to Und-P is then flipped across the IM by WzxE (26). The ECA chains are polymerized by WzyE (27), and the chain length is controlled by WzzE (28). Three forms of ECA are made from polymerized ECA chains. In the first, LPS-linked ECA (ECA_LPS_), the ECA chain is transferred to the core sugar moiety of LPS by WaaL (29), the same gene responsible for attaching O antigen to core, and the molecule is transferred to the cell surface, presumably by the Lpt system. In the second, phosphatidylglycerol-linked ECA (ECA_PG_), the ECA chain is attached to phosphoglyceride by a phosphodiester linkage (30) and the molecule is surface exposed through an unknown pathway (31, 32). In the third form, cyclic ECA (ECA_CYC_), an ECA chain of a precise chain length (4 to 6, depending on species) is cyclized in a reaction dependent on WzzE (33–35). This molecule remains in the periplasm (34).

10.1128/mBio.01321-18.1 FIG S1  Overview of ECA biosynthesis. (A) ECA biosynthesis begins with the transfer of GlcNAc-1-phosphate to Und-P by WecA. (B) Subsequently, WecB and WecC synthesize the ManNAcA that is attached to Und-P-P-GlcNAc by WecG. (C) Fuc4NAc is synthesized by four members of the ECA pathway, although two steps are redundant with those of the O-antigen biosynthesis pathway. Fuc4NAc is attached to Und-P-P-GlcNAc-ManNAcA by WecF, and the molecule is flipped across the inner membrane by WzxE. (D) ECA subunits are polymerized by WzyE, with the length of the final chain controlled by WzzE, the chain length regulator. The polymerized ECA chain can be made into three types of ECA: ECA_LPS_, ECA_PG_, and ECA_CYC_. (E) To synthesize ECA_LPS_, the ECA chain is transferred to the core of an LPS molecule by WaaL, the O-antigen ligase. This molecule is presumed to traffic to the outer membrane through the Lpt pathway. (F) To synthesize ECA_PG_, the ECA chain is transferred to phosphoglyceride through an unknown mechanism and then transferred to the cell surface through an unknown pathway. (G) ECA_CYC_ is removed from Und-P-P through cyclization and the cyclized molecule remains in the periplasm. Synthesis of ECA_CYC_ requires a precise chain length (4 in E. coli) and so cannot proceed without WzzE. Download FIG S1, EPS file, 2.7 MB. Copyright © 2018 Mitchell et al. 2018 Mitchell et al. This content is distributed under the terms of the Creative Commons Attribution 4.0 International license.

Previously, we investigated changes to the Escherichia coli K-12 OM that occur during growth under different nutrient conditions (36) and determined that an RpoS-dependent mechanism strengthens the envelope permeability barrier under carbon-limiting conditions in a manner that depends on the presence of the genes for several proteins. Of these, YhdP is a large protein of unknown function that is predicted to be located in the IM with the majority of the protein exposed to the periplasm (37). Unlike our other hits, *yhdP* deletion (*ΔyhdP*) has strong phenotypes regardless of growth phase. In fact, in a large-scale study on the effects of gene deletions on chemical sensitivity (38), *yhdP* scored second highest for SDS EDTA (sodium dodecyl sulfate, ethylenediaminetetraacetic acid) sensitivity as well as in the top 15 hits for vancomycin sensitivity. Despite the high level of envelope permeability caused with deletion of *yhdP*, the function of YhdP is completely unknown. In addition, *yhdP* appears to be restricted phylogenetically (39), suggesting that it may play a role that is specific to a subset of species.

Here, we demonstrate that mutations that block ECA biosynthesis restore the envelope permeability barrier of Δ*yhdP* strains. Furthermore, we demonstrate that *yhdP*, which phylogenetically co-occurs with ECA biosynthesis genes, directly or indirectly controls ECA levels. We were able to trace the suppression specifically to the removal of ECA_CYC_ and demonstrate that, even in a wild-type background, removing ECA_CYC_ changes the OM permeability barrier. Therefore, ECA_CYC_ plays a role in maintaining the OM permeability barrier, and its activity is regulated by YhdP.

## RESULTS

### Deletion of *yhdP* causes OM permeability.

**FIG 1:**
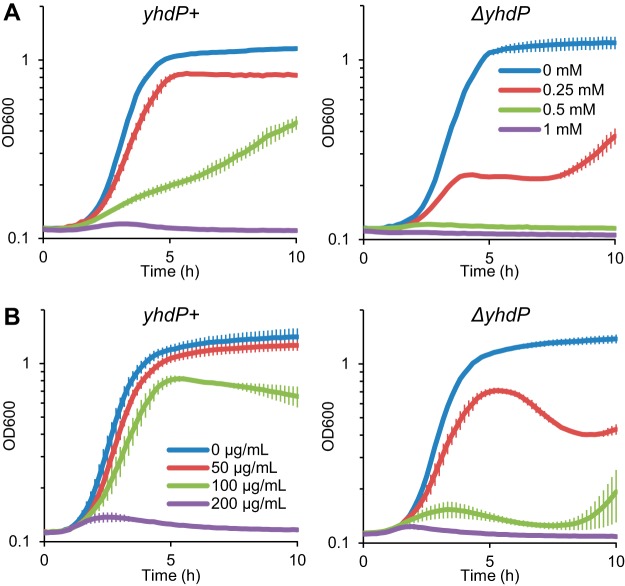
Deletion of *yhdP* causes SDS EDTA and vancomycin sensitivity. (A) Cells with wild-type *yhdP* or from a *ΔyhdP* deletion mutant were diluted into fresh media containing 0.05% SDS and the indicated concentration of EDTA, and growth was assayed based on the OD_600_ every 10 min. The *ΔyhdP* strain was more sensitive to EDTA in the presence of SDS than the *yhdP*^*+*^ strain. (B) Cells were grown as described for panel A, with the indicated concentration of vancomycin. The *ΔyhdP* strain lysed at lower concentrations of vancomycin than those that affected the *yhdP*^*+*^ strain. Data are averages of three independent biological replicates ± the SEM on a log_10_ scale.

Our initial screen identifying *yhdP* was based on sensitivity to SDS treatment during stationary phase, and we also found that *ΔyhdP* caused sensitivity to 2% SDS in actively growing cells (36); therefore, we treated *yhdP*^*+*^ and *ΔyhdP* cells with SDS and increasing concentrations of EDTA and measured their growth. EDTA disrupts the bridging of LPS molecules by divalent cations, sensitizing the OM to the presence of detergents and allowing for the detection OM defects (40). Both *yhdP*^*+*^ and *ΔyhdP* cells grew to stationary phase with 0.05% SDS alone; however, a low concentration of EDTA (0.25 mM) caused a large growth defect in *ΔyhdP* cells, while causing a minimal effect on *yhdP*^*+*^ cells (Fig. 1A). In addition, a higher concentration of EDTA (0.5 mM) completely impaired the growth of *ΔyhdP* cells, while still allowing for growth of *yhdP*^*+*^ cells. These data suggest that there is a change in outer membrane structure when YhdP is removed, and so we investigated whether *ΔyhdP* causes permeability to other toxic agents.

Disk assay results suggested that *ΔyhdP* cells might be sensitive to vancomycin. Vancomycin is a glycopeptide antibiotic that targets peptidoglycan biosynthesis, which is commonly used to treat antibiotic-resistant Gram-positive infections but is largely incapable of traversing the Gram-negative OM (41). Permeability of the OM to vancomycin is thought to be caused by “cracks” between patches of phospholipids and LPS (42). We analyzed growth curves of *yhdP*^*+*^ and *ΔyhdP* cells with increasing dosages of vancomycin. Similarly to *ΔyhdP*’s SDS EDTA sensitivity, a low dose of vancomycin (50 µg/ml) caused lysis of *ΔyhdP* cells while not affecting *yhdP*^*+*^ growth (Fig. 1B). A higher dose of vancomycin (100 µg/ml) completely inhibited growth of *ΔyhdP* cells while only minimally affecting *yhdP*^*+*^ cells. In fact, the increase in vancomycin sensitivity with *ΔyhdP* can also be observed by a lowering of the vancomycin MIC (MIC) for this strain (Fig. S2). Both the increased vancomycin and SDS EDTA sensitivity of *ΔyhdP* cells suggest that there is a change in OM structure in this mutant that leads to increased permeability.

10.1128/mBio.01321-18.2 FIG S2  Deletion of *yhdP* lowers the vancomycin MIC. Cells with either wild-type *yhdP* or with *yhdP* deleted were diluted to a very low cell density and then incubated overnight with 2-fold serial dilutions of vancomycin. The OD_600_ was used to assay growth. The lowest concentration at which no growth was observed was considered the MIC. Data are the average results of three biological replicates ± the SEM. Download FIG S2, EPS file, 1.1 MB. Copyright © 2018 Mitchell et al. 2018 Mitchell et al. This content is distributed under the terms of the Creative Commons Attribution 4.0 International license.

### Disrupting ECA biosynthesis suppresses envelope permeability in *ΔyhdP* strains.

**FIG 2:**
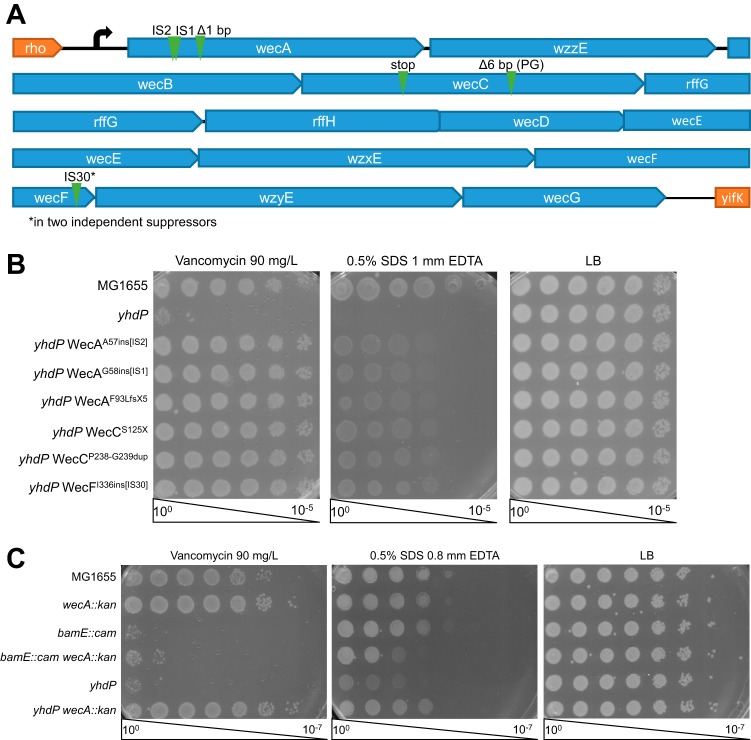
Loss-of-function mutations in ECA biosynthesis suppress *ΔyhdP*. Screening for suppressors of *ΔyhdP* vancomycin and SDS EDTA sensitivity led to the isolated seven suppressor mutations. (A) The locations of the suppressing mutations, all of which map to the *wec* operon, are shown. IS, native insertion sequence. (B) Efficiency of plating assays (EOPs) were performed by plating serial dilutions of the indicated culture on LB plates with the indicated additions to gauge to what degree the suppressor mutants suppressed the *ΔyhdP* strain's phenotypes. Complete suppression of vancomycin sensitivity and almost complete suppression of SDS EDTA sensitivity were observed. (C) EOPs were performed to determine whether suppression of vancomycin and SDS EDTA sensitivity by loss-of-function mutations in the *wec* operon was universal. Deletion of *wecA* suppressed the *ΔyhdP* strain but not deletion of *bamE*, suggesting the suppression is specific to *yhdP*. EOP images are representative of three independent experiments.

As no functions are known for YhdP, we then sought to isolate mutations suppressing strain *ΔyhdP*’s envelope permeability defects in order to determine in what pathway YhdP might be working. The slight growth we observed late after SDS EDTA and vancomycin treatment (Fig. 1) suggested that spontaneous suppressor mutants are common within *ΔyhdP* strain cultures. Therefore, we plated *ΔyhdP* cells on a concentration of vancomycin at which growth of these cells is inhibited but *yhdP*^*+*^ cells could grow, and we isolated spontaneous suppressor mutants that were capable of growth on this medium. Then, we conducted a secondary screen of these suppressors to identify those that restored both vancomycin and SDS EDTA resistance. We isolated seven spontaneous suppressors that restored both phenotypes, all of which mapped to the ECA biosynthesis (*wec*) operon. All seven appeared to be loss-of-function alleles (Fig. 2A and B).

We then asked whether suppression of the *ΔyhdP* strain envelope permeability by disruption of the *wec* operon is specific to *yhdP* or is a general mechanism of vancomycin and SDS EDTA resistance. To answer this question, we utilized a deletion allele of *bamE*, a component of the β-barrel assembly machine responsible for folding outer membrane proteins (OMPs) into the outer membrane (43). Removal of this nonessential lipoprotein from the complex leads to a similar level of vancomycin sensitivity as *ΔyhdP* and causes slight SDS EDTA sensitivity (44, 45). We combined this deletion with a deletion of *wecA*, which is responsible for the addition of the first sugar in ECA to Und-P (46). Some vancomycin resistance was caused by deletion of *wecA* alone (Fig. 2C). When combined with a deletion of *bamE*, *wecA* deletion caused only minimal suppression of vancomycin sensitivity and worsened the *bamE* SDS EDTA sensitivity. In contrast, *wecA* deletion fully suppressed both the vancomycin and SDS EDTA sensitivities of a *ΔyhdP* strain. These data demonstrate that disruption of ECA biosynthesis is not a universal suppressor of vancomycin and SDS EDTA sensitivity and suggest that this suppression is specific to the *ΔyhdP* strain.

We conducted a transposon mutagenesis screen in an effort to identify additional suppressors of the *ΔyhdP* mutant strain envelope permeability. Briefly, we identified vancomycin-resistant clones from a pool of 10,000 transposon mutants in a *ΔyhdP* strain, mapped the transposon insertion sites in these mutants, and conducted a secondary screen for SDS EDTA resistance (Fig. S3A). The only mutations we identified that suppressed both the vancomycin and SDS EDTA phenotypes were in the *wec* operon (Table S1); furthermore, we identified mutations in every gene in the *wec* operon except those that are redundant with O-antigen biosynthesis genes (*rffG*, *wzxE*) (34, 47) and *wzyE*, for which disruption is toxic (34) (Fig. S3B). Given that the *wec* genes form an operon, it is likely that some of these insertions may be polar. With more than 2× genome coverage, *wec* operon mutations were the only mutations to suppress both of *ΔyhdP*’s phenotypes, suggesting that our suppressor screen may be saturated.

10.1128/mBio.01321-18.3 FIG S3  Transposon screen identified insertions in ECA biosynthesis genes as suppressors of the *ΔyhdP* strain's membrane permeability. (A) To identify loss-of-function mutations that suppress the *ΔyhdP* strain's phenotypes, a transposon library was generated in a *ΔyhdP* strain background and selected for vancomycin resistance. The transposon mutations from the isolated mutants were transferred to new strains to verify that the vancomycin suppression was linked to the insertion of the transposon. Then, the isolated strains were tested for SDS EDTA resistance and the transposon insertion sites were mapped by arbitrary PCR. (B) The genomic locations of the insertions suppressing both vancomycin and SDS EDTA sensitivity are shown. All of these insertions mapped to the *wec* operon. Download FIG S3, EPS file, 1.3 MB. Copyright © 2018 Mitchell et al. 2018 Mitchell et al. This content is distributed under the terms of the Creative Commons Attribution 4.0 International license.

10.1128/mBio.01321-18.9 TABLE S1  Phenotypes of insertions isolated from the transposon screen. Download TABLE S1, DOCX file, 0.01 MB. Copyright © 2018 Mitchell et al. 2018 Mitchell et al. This content is distributed under the terms of the Creative Commons Attribution 4.0 International license.

### Loss of ECA is directly responsible for the suppression of the *ΔyhdP* strain.

Because the ECA biosynthetic pathway interacts with the biosynthesis pathway for peptidoglycan and other extracytoplasmic glycans, disruption of ECA biosynthesis can cause cellular changes that are more wide-ranging than simple removal of ECA. Specifically, O-antigen biosynthesis, peptidoglycan biosynthesis, and ECA biosynthesis all compete for both precursor sugar molecules and for the lipid carrier on which the molecules are assembled, Und-P. Although our strains are O-antigen negative, when intermediate steps in the ECA biosynthesis pathway are disrupted, ECA intermediates (aminoglycans linked to Und-P-P) accumulate and sequester Und-P, stressing the peptidoglycan biosynthesis pathway (10). In contrast, when the first step in ECA biosynthesis (catalyzed by WecA) (46) is prevented, the pool of sugar precursors and Und-P available for peptidoglycan synthesis is increased. Therefore, we sought to determine whether (i) the phenotypes of *ΔyhdP* mutant cells were caused by Und-P stress and (ii) whether relieving Und-P stress suppresses the *ΔyhdP* strain.

**FIG 3:**
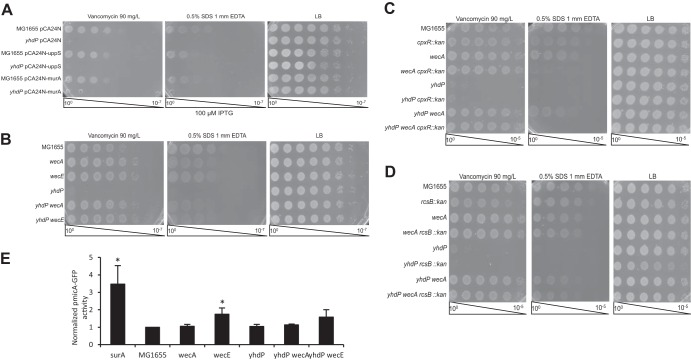
Suppression does not relate to Und-P availability or stress responses. (A) To determine whether the *ΔyhdP* strain is suppressed by relieving Und-P stress, EOPs were performed on strains carrying the indicated overexpression constructs. Overexpression of *uppS* and *murA* did not suppress the *ΔyhdP* strain, demonstrating that the *ΔyhdP* strain's phenotypes are not caused by effects of Und-P stress on peptidoglycan. (B) EOPs were performed to determine whether disruptions in ECA biosynthesis that increased Und-P availability for peptidoglycan synthesis (*ΔwecA*) and that decreased Und-P availability for peptidoglycan synthesis (*ΔwecE*) both suppress the *ΔyhdP* strain phenotypes. Both of these mutations suppressed the *ΔyhdP* strain phenotypes to an equal extent, suggesting that suppression is unrelated to Und-P availability. (C) EOPs were performed to determine whether the Cpx response was responsible for suppression of the *ΔyhdP* strain's phenotypes by disruptions of ECA biosynthesis. Suppression was observed with *wecA* deletion, even in the presence of *cpxR* deletion, demonstrating that the Cpx response is not necessary for suppression. (D) EOPs were performed to determine whether the Rcs response was required for suppression. Suppression was observed in the presence of *rcsB* deletion, demonstrating that the Rcs response is not necessary for the suppression of the *ΔyhdP* strain's phenotypes. EOPs images are representative of three independent experiments. (E) Activity of a σ^E^ reporter was assayed to determine whether suppression of the *ΔyhdP* strain correlated with σ^E^ activation. Suppression of the *ΔyhdP* strain's phenotypes did not correlate with σ^E^ activation, suggesting this is not the mechanism of suppression. Data shown are the average results of three independent biological replicates ± the SEM. Significance was calculated using the Mann-Whitney test. *, *P* < 0.05 compared to the appropriate parent strain (MG1655 or *ΔyhdP*).

Stress on the pool of Und-P has previously been detected using linkage disruption with a marker linked to *mrcB*::*kan* (48). As *mrcB* (PBP1B) is important for transglycosylation and transpeptidation of peptidoglycan precursors (49), deletion of *mrcB* in a strain with stress on the pool of available Und-P causes significant toxicity and can be synthetically lethal. This causes disruption of the linkage between the Tn*10* marker and the *mrcB* deletion (i.e., fewer colonies with the Tn*10* marker have received the *mrcB* deletion). However, we detected no linkage disruption with the *ΔyhdP* strain in the presence or absence of *wecA* (Fig. S4A), suggesting that the *ΔyhdP* mutant does not cause lipid carrier stress. To relieve possible Und-P stress, we overexpressed *uppS*, responsible for synthesizing Und-P, and *murA*, responsible for the first committed step in peptidoglycan synthesis (50, 51). Overexpression of these genes has previously been shown to relieve peptidoglycan stress caused by Und-P availability (48, 52). Overexpression of these genes had no effect on envelope permeability in a *ΔyhdP* background (Fig. 3A). Overexpression of *uppP*, a gene responsible for recycling Und-P (53), and *mrcB*, the gene encoding PBP1B, also had no effect on the *ΔyhdP* strain's envelope permeability (Fig. S4B). These data show that the phenotypes of the *ΔyhdP* deletion mutant are not caused by Und-P stress.

10.1128/mBio.01321-18.4 FIG S4  Membrane permeability in Δ*yhdP* strains is not caused by Und-P stress. (A) To determine whether *ΔyhdP* causes Und-P stress, the P1 transduction linkage between a Tn*10* marker and *mrcB*::*kan* was analyzed. Briefly, cells of the indicated genotype were transduced with a P1*vir* lysate carrying *zad-220*::Tn*10 mrcB*::*kan* and selected for tetracycline resistance. Resistant colonies were replicate plated to kanamycin plates to determine the presence of the *mrcB*::*kan* allele. The data shown indicate the percentages of transductants carrying the Tn*10* marker that also received the *mrcB*::*kan* allele in the indicated strain background. All percentages were calculated from >150 transductants from two separate transductions. (B) To determine whether relieving Und-P stress can suppress the *ΔyhdP* strain, EOPs were performed with strains overexpressing the indicated genes with the highest level of induction that did not cause toxicity. Neither overexpression of *uppP* nor *mrcB* suppressed the *ΔyhdP* strain's phenotypes, suggesting that these phenotypes are not caused by Und-P stress. EOP images represent results of three independent experiments. Download FIG S4, JPG file, 0.3 MB. Copyright © 2018 Mitchell et al. 2018 Mitchell et al. This content is distributed under the terms of the Creative Commons Attribution 4.0 International license.

To verify further that the mechanism of *ΔyhdP* strain suppression by disruption of the ECA biosynthesis operon was not through effects on Und-P, we asked whether deletion of the first gene in ECA synthesis, *wecA*, and deletion of a gene in an intermediate step of ECA synthesis, *wecE*, would have to the same effects on envelope permeability in a *ΔyhdP* strain. Deletions of both *wecA* and *wecE* caused slight vancomycin resistance and SDS EDTA sensitivity in a *yhdP*^*+*^ background; however, when they were combined in the *ΔyhdP* deletion strain, the vancomycin and SDS EDTA resistance were both restored to the level of the ECA mutants alone (Fig. 3B). As both *ΔwecA* and *ΔwecE* mutant strains fully suppress the envelope permeability defects of a *ΔyhdP* strain despite having opposite effects on availability of Und-P and precursors, these data demonstrate that the suppression for the *ΔyhdP* strain is not due to modification of the peptidoglycan biosynthesis pathway.

Because of peptidoglycan defects and the accumulation of Und-P-linked ECA precursors, disruption of the ECA operon can also activate the Cpx, Rcs, and σ^E^ stress responses (10, 11). In fact, in Serratia marcescens, even disruption of *wecA* can activate the Rcs response (12). Therefore, we tested whether activation of stress responses was responsible for suppression of the *ΔyhdP* strain's envelope permeability by disruption of the ECA biosynthesis operon. The Cpx and Rcs stress responses are nonessential and their activity can be prevented by removal of their response regulators, CpxR and RcsB, respectively (54, 55). Disruption of *cpxR* has no effect on the suppression of the *ΔyhdP* strain's vancomycin sensitivity by *ΔwecA*, although synthetic SDS EDTA sensitivity in Δ*wecA* Δ*cpxR* double mutants prevents assessment of the role of Cpx on SDS EDTA sensitivity caused by *yhdP* deletion (Fig. 3C). Disruption of *rcsB* has no effect on the suppression of either the *ΔyhdP* strain's vancomycin or SDS EDTA sensitivity by the *wecA* deletion (Fig. 3D). These data demonstrate that neither the Cpx nor the Rcs stress response is necessary for the suppression of the *ΔyhdP* strain's phenotypes by the disruption of ECA biosynthesis. Although the σ^E^ response is essential in E. coli (56), the activation of the σ^E^ response can be monitored using reporters linked to σ^E^-responsive promoters. One such reporter consists of the *micA* promoter, driving expression of green fluorescent protein (GFP) (57). Using this reporter, we found that activation of the σ^E^ response by ECA operon disruptions was not necessary for these disruptions to suppress the *ΔyhdP* strain's envelope permeability (Fig. 3E). These data together with the rarity of other suppressing mutations for *ΔyhdP* strongly suggest that YhdP is functionally connected with ECA.

### The genes for YhdP and ECA occur in the same genomes.

As our data suggested that ECA and YhdP may interact and ECA is restricted to *Enterobacteriaceae*, we examined the phylogenetic distribution of *yhdP*. We used STRING-DB (39) to search for possible homologues of *yhdP* and to score the homology of the detected genes. The vast majority of the homologues for *yhdP* were found to be in *Enterobacteriaceae*. In fact, homologues of YhdP outside of *Enterobacteriaceae* are only detected in some other Gammaprotobacteria and some Betaprotobacteria; however, none of the YhdP homologues detected for YhdP outside of *Enterobacteriaceae* had a higher homology with E. coli K-12 YhdP than a possible homologue in Indian rice (Oryza sativa Indica) (Fig. S5A). As YhdP is part of a family of proteins, the AsmA family (58), the *yhdP* homologues detected outside *Enterobacteriaceae* possibly represent other members of the AsmA family.

10.1128/mBio.01321-18.5 FIG S5  YhdP is restricted to *Enterobacteriaceae*. (A) The STRING database was used to calculate homology scores for possible YhdP homologues inside and outside *Enterobacteriaceae*. The range of homology scores within a genus, class, or organism is shown with black markers. Gray dots indicate the presence of species in which no homologue was detected. *Enterobacteriaceae* are separated from other groups by a solid line. The score of a possible homologue detected in a eukaryotic organism is depicted as a hashed line. Only possible homologues in *Enterobacteriaceae* score higher than the possible eukaryotic homologue. (B) The STRING database was used to calculate phylogenetic co-occurrence scores for the indicated pairs of genes, indicating the frequency with which genomes have both genes and suggesting a functional relationship. A high level of co-occurrence was observed between *yhdP* and ECA biosynthesis genes, in some cases higher than that observed between ECA biosynthesis genes. Download FIG S5, EPS file, 1.7 MB. Copyright © 2018 Mitchell et al. 2018 Mitchell et al. This content is distributed under the terms of the Creative Commons Attribution 4.0 International license.

To examine further the distribution of the ECA biosynthetic genes and *yhdP*, we used STRING-DB (39) to calculate phylogenetic co-occurrence scores based on genes with homology found across genomes. The three genes within the *wec* operon whose products form a complex to flip ECA to the outer leaflet of the IM (*wzxE*), polymerize ECA (*wzyE*), and control ECA chain length (*wzzE*) have phylogenetic co-occurrence scores with each other of 0.70 to 0.78 (Fig. S5B). The co-occurrence scores for these genes with *yhdP* range from 0.40 to 0.76, which are within the range of the co-occurrence scores for pairs of genes within the *wec* operon (0.15 to 0.78). The highest co-occurrence pair for *yhdP* was found with *wzzE*. These data demonstrate that genomes containing the machinery to make ECA also contain *yhdP*.

### YhdP changes ECA levels.

**FIG 4:**
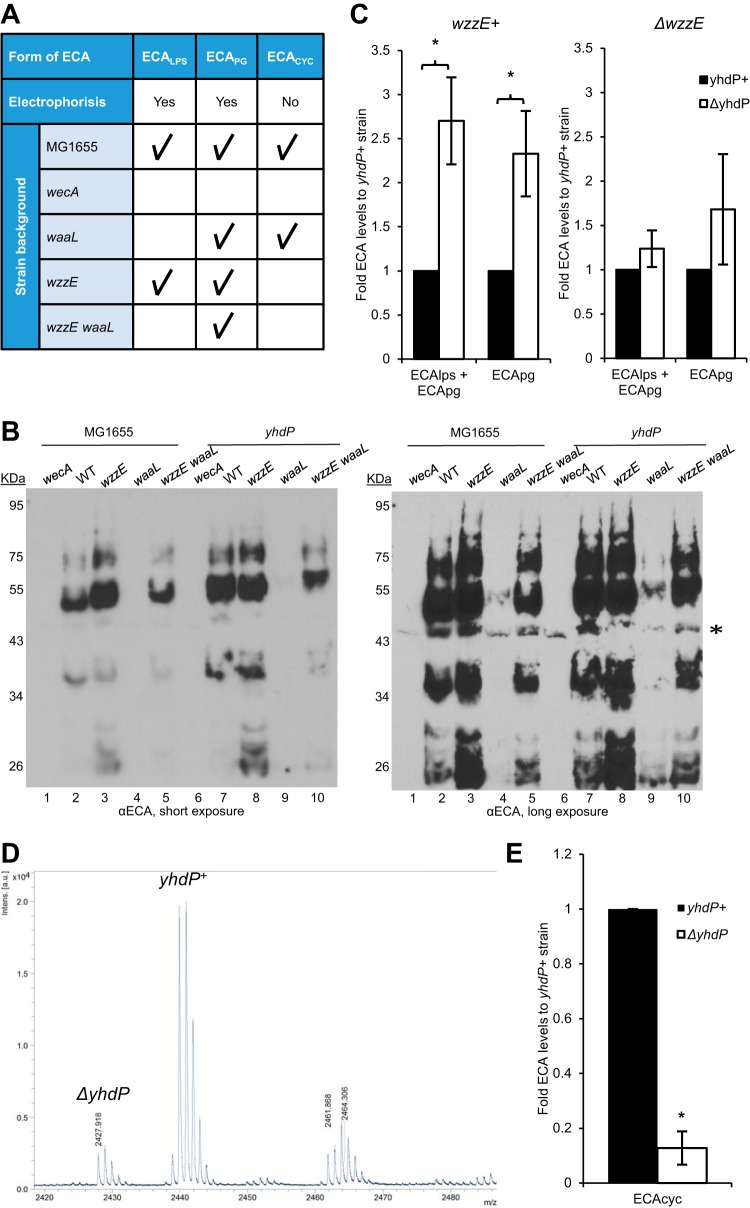
YhdP interacts genetically with ECA. (A) Distinctions between forms of ECA are shown in table form, including whether they can be subjected to PAGE analysis and immunoblotting (“electrophoresis”) and which forms are present in genetic backgrounds with the indicated gene deletions. The presence of the form of ECA is indicated with a check mark. (B) Immunoblot analysis was performed for strains with the indicated gene deletions and probed with anti-ECA antibody to assay changes to ECA caused by the *yhdP* deletion. Strains with no mutations other than *yhdP*^*+/−*^ are indicated by WT. Bands from low molecular weights to high molecular weights represent increasing ECA chain lengths. The types of ECA that can be observed in each genetic background are detailed in Fig. 4A. Short and long exposures are shown. *, a nonspecific band. All other bands are forms of ECA. Images are representative of five independent experiments. (C) Densitometry was performed from immunoblots to quantitate levels of the indicated types of ECA in the given background. The levels of membrane-bound ECA were found to be higher with the *ΔyhdP* strain only when *wzzE* was present. Fold values compared to the *yhdP*^*+*^ strain are shown as the average of three to five independent experiments ± the SEM. (D) Levels of ECA_CYC_ were analyzed by MALDI-TOF with relative values comparing *yhdP*^*+*^ cells labeled with ^15^N to *ΔyhdP* cells. Cells were combined before purification to allow for direct comparison of levels of heavy ECA_CYC_ (*m/z* 2,440) and normal ECA_CYC_ (*m/z* 2,428). A representative image is shown and normal and heavy peaks are labeled by their originating strain. The unlabeled higher-molecular-weight species is a modified form of ECA_CYC_. (E) Quantification of ECA_CYC_ levels is shown as average relative levels from three biological replicates ± the SEM. Levels of ECA_CYC_ were lowered in a strain *ΔyhdP* background. *, *P* < 0.05 compared to the *yhdP*^+^ strain.

Given that YhdP and ECA are functionally related, we investigated whether deletion of *ΔyhdP* causes changes to ECA abundance or chain length. There is no apparent change in the surface exposure of ECA_PG_ or ECA_LPS_ in the absence of *yhdP* (Fig. S6). By immunoblotting, we detected ECA_LPS_ and ECA_PG_ and compared the levels and chain length with and without *yhdP* (Fig. 4B). We observed a range of bands with the lowest molecular weight band likely indicating molecules with one repeat unit of ECA and higher molecular weight bands indicating molecules with more repeat units of ECA. Lanes 2 and 7 show a combination of ECA_LPS_ and ECA_PG_ due to the wild-type genetic background, while lanes 4 and 9 (*ΔwaaL* strains) show ECA_PG_ alone, as ECA_LPS_ cannot be produced in these strains (Fig. 4A). Given the lack of bands with Δ*wecA* samples where there is no ECA, all bands other than the band designated with an asterisk were taken to be ECA. Due to the use of a polyclonal antibody, the levels of ECA_LPS_ and ECA_PG_ cannot be directly compared.

10.1128/mBio.01321-18.6 FIG S6  YhdP does not affect the surface exposure of ECA_LPS_ or ECA_PG_. Cells of the indicated strains were spun down and resuspended in phosphate-buffered saline and either left at room temperature (whole) or boiled (lysates). Samples were then spotted on membranes, and immunoblot analysis was performed with the indicated antibodies. BamD served as a negative control for surface exposure, while RcsF served as a positive control for surface exposure. Lane 1 served as a negative control for ECA. Lanes 2 and 4 may have both ECA_LPS_ and ECA_PG_ surface exposed, while lanes 3 and 5 only make ECA_PG_. The absence of *yhdP* does not affect whether these forms of ECA are surface exposed. Download FIG S6, EPS file, 1.8 MB. Copyright © 2018 Mitchell et al. 2018 Mitchell et al. This content is distributed under the terms of the Creative Commons Attribution 4.0 International license.

There was no apparent difference in chain length between *yhdP*^*+*^ and *ΔyhdP* strains (lanes 2 and 7); however, levels of ECA_LPS_ and ECA_PG_ together (lanes 2 and 7) were higher in a *ΔyhdP* strain than in a *yhdP*^*+*^ strain. This is also true for ECA_PG_ alone (lanes 4 and 9). Interestingly, this 2- to 3-fold increase in ECA levels only occurred in the presence of *wzzE* (Fig. 4C). The reason for the large increase in ECA_PG_ levels between the *ΔwaaL* and *ΔwzzE ΔwaaL* strains remains an interesting question for further investigation.

Unlike the lipid-linked forms of ECA, ECA_CYC_ is not charged and cannot be detected by immunoblotting (Fig. 4A). Instead, we utilized a quantitative MALDI-TOF (matrix-assisted laser desorption ionization-time of flight) approach to detect and quantitate ECA_CYC_ in purified samples. By examining the *m/z* ratios of ECA_CYC_ peaks, which are present in *wzzE*^*+*^ strains and absent in *ΔwzzE* strains (Fig. 4A), we determined that the cyclization and nonstoichiometric acetylation of ECA_CYC_ were not changed in a *ΔyhdP* strain (Fig. S7A). Therefore, to quantitate ECA_CYC_ levels, we utilized *ΔwecH* strains that do not acetylate ECA and that do not affect *ΔyhdP* phenotypes to minimize the number of ECA_CYC_ peaks. We then grew *yhdP*^*+*^ strains with a nitrogen source containing ^15^N and *ΔyhdP* cells with a nitrogen source containing ^14^N. This shifted the *m/z* ratio of the ECA_CYC_ by 12, as ECA_CYC_ contains 12 nitrogen atoms, and allowed the comparison of the *yhdP*^*+*^ and *ΔyhdP* strains' ECA_CYC_ on the same spectra (Fig. S7B).

10.1128/mBio.01321-18.7 FIG S7  Cyclic ECA levels are lowered in Δ*yhdP* strains. (A) To detect possible changes in ECA_CYC_ structure (e.g., modified cyclization, changes to acetylation), MALDI-TOF analysis was performed on purified ECA_CYC_ from the indicated strains. Tracings are shown in the indicated colors. The inset graph shows ratios of ECA_CYC_ peaks, all of which were absent in *ΔwecA* and *ΔwzzE* mutant strains. No changes in the ECA_CYC_ structure were apparent with the *ΔyhdP* strain. (B) To detect levels of ECA_CYC_, *wecH*::*kan* strains were utilized to prevent nonstoichiometric acetylation of ECA, and cultures were grown in minimal media with a normal (*ΔyhdP*) or a heavy (*yhdP*^*+*^) nitrogen source to shift the molecular mass of ECA_CYC_ by 12 Da. These cultures were either combined before purification of ECA_CYC_ or purified separately to verify *m/z* readings for the ECA_CYC_ species. The samples were assayed by MALDI-TOF. Representative tracings of the peaks detected by MALDI-TOF in the size range of ECA_CYC_ are shown for individual control samples as well as for a sample for quantitation containing both normal (*ΔyhdP*) and heavy (*yhdP*^*+*^) ECA_CYC_. The *m/z* values for normal and heavy ECA_CYC_ were as expected (2,428 and 2,440, respectively). Levels of ECA_CYC_ in *ΔyhdP* strain cells were lower than levels in *yhdP*^*+*^ cells. *ΔwzzE* cells represent a negative control. Download FIG S7, EPS file, 2.5 MB. Copyright © 2018 Mitchell et al. 2018 Mitchell et al. This content is distributed under the terms of the Creative Commons Attribution 4.0 International license.

To quantitate relative ECA_CYC_ levels, we combined *yhdP*^*+*^ cells grown with ^15^N with an equal number of *ΔyhdP* cells grown with ^14^N as one sample before purification of ECA_CYC_, allowing direct comparison of the peaks generated from each strain. This approach indicated that levels of ECA_CYC_ are decreased in the *ΔyhdP* strain (Fig. 4D). Over several biological replicates, we found the decrease in the *ΔyhdP* strain to be almost 8-fold (Fig. 4E). The phylogenetic co-occurrence of *yhdP* with ECA biosynthesis genes and the changes to ECA in the absence of YhdP provides further evidence that YhdP plays a role related to ECA.

### YhdP prevents ECA_CYC_ from damaging the OM permeability barrier.

Given that the different forms of ECA are present in different cellular compartments and presumably play different roles, we then asked which form of ECA is responsible for the *ΔyhdP* strain's phenotypes. We hypothesized that one of the membrane-associated forms of ECA would be responsible for the permeability defects, as these molecules are part of the OM and the levels of these molecules are increased when YhdP is removed. We tested this hypothesis by removing specific forms of ECA and determining whether the *ΔyhdP* strain's envelope permeability was suppressed.

**FIG 5:**
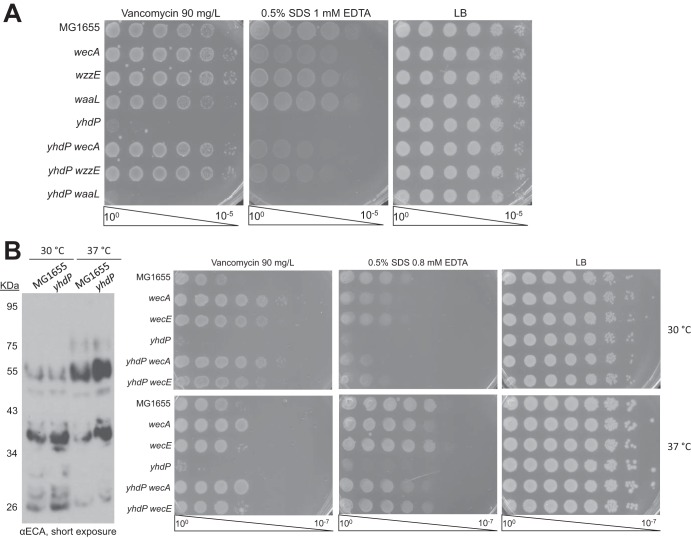
Antibiotic sensitivity from the *ΔyhdP* strain is mediated by a functional interaction with ECA_CYC_. (A) To determine what form of ECA is important for causing the *ΔyhdP* strain's phenotypes, EOPs were performed on strains with the indicated deletions. Suppression by *wzzE* deletion and lack of suppression by *waaL* deletion suggest that ECA_CYC_ may be responsible for the *ΔyhdP* strain's phenotypes. (B) To eliminate the possibility that suppression in a *ΔwzzE* deletion strain occurs through changes in ECA chain length, EOPs were performed at 30°C and at 37°C to assay the *ΔyhdP* strain's phenotypes. Immunoblot analysis was used to determine ECA chain length at these temperatures. The model chain length of ECA was found to be four at 30°C and six at 37°C. However, the *ΔyhdP* strain has strong phenotypes at both temperatures, suggesting that changes in chain length are not responsible for suppression. All images are representative of three independent experiments.

We compared *wecA* deletion, which removes all forms of ECA (46) and suppresses, with *waaL* deletion, which specifically prevents the formation of ECA_LPS_ (29), and with *wzzE* deletion, which prevents the formation of ECA_CYC_ but allows the formation of ECA_LPS_ and ECA_PG_, albeit with random chain length (28, 34). Currently, there is no way to remove ECA_PG_ without removing the other forms of ECA. We observed full suppression of the *ΔyhdP* strain's envelope permeability with both the *wecA* and *wzzE* deletions; however, *waaL* deletion had no effect on the *ΔyhdP* strain's phenotypes (Fig. 5A). These data demonstrate that ECA_LPS_ does not contribute to the *ΔyhdP* strain's phenotypes. The suppression of the *ΔyhdP* strain by *wzzE* deletion did not rely on stress response activation (Fig. S8).

10.1128/mBio.01321-18.8 FIG S8  Suppression of the *ΔyhdP* strain's envelope permeability by *wzzE* deletion is not dependent on stress responses. (A) To determine whether activation of the Cpx stress response is responsible for suppression of the *ΔyhdP* strain's phenotypes by *wzzE* deletion, EOPs were performed with strains carrying a *cpxR*::*kan* allele. (B) To determine whether activation of the Rcs stress response is for suppression of the *ΔyhdP* strain by *wzzE* deletion, EOPs were performed with *rcsB*::*kan* strains. Deletion of neither *cpxR* nor *rcsB* prevented suppression by *wzzE* deletion. EOPs images are representative of three separate experiments. (C) To determine whether *wzzE* deletion activates σ^E^, the activity of a σ^E^ Reporter was assayed in the indicated strains. Data for *wzzE* nondeletion strains were identical to those shown in Fig. 3D. Deletion of *wzzE* did not activate the σ^E^ stress response. The fold changes of reporter activity compared to the wild-type strain are the averages of three independent experiments ± the SEM. Download FIG S8, JPG file, 0.3 MB. Copyright © 2018 Mitchell et al. 2018 Mitchell et al. This content is distributed under the terms of the Creative Commons Attribution 4.0 International license.

WzzE is the chain length regulator for ECA (28) and its removal causes several changes to ECA, some of which can be observed in Fig. 4B (compare lanes 2 and 3 and lanes 4 and 5): (i) the amount of ECA_LPS_ and ECA_PG_ is increased; (ii) more short (less than 6 copies) and long (more than 7 copies) ECA chains are made; (iii) no ECA_CYC_ is produced (see Fig. S7). Given that completely removing ECA suppresses the *ΔyhdP* strain's phenotypes and *yhdP* deletion increases levels of ECA_LPS_ and ECA_PG_, we find it unlikely that the mechanism through which *wzzE* deletion suppresses the *ΔyhdP* strain's phenotypes is through further increasing these levels. To investigate the possibility that increasing or decreasing ECA chain length suppresses the *ΔyhdP* strain, we tested the *ΔyhdP* strain's envelope permeability and suppression at 30°C, where the modal chain length of ECA appears to be four copies; at 37°C the modal chain length of ECA is six to seven copies (Fig. 5B). Although intrinsic sensitivities to SDS EDTA and vancomycin differ between these temperatures, neither temperature suppresses the *ΔyhdP* strain's phenotypes, nor does the suppression of the *ΔyhdP* strain occur by preventing ECA synthesis change. Thus, despite the fact that *yhdP* deletion lowered ECA_CYC_ levels, preventing ECA_CYC_ synthesis suppressed the envelope permeability of *ΔyhdP* strains, demonstrating a specific, functional interaction between YhdP and ECA_CYC_.

### ECA_CYC_ maintains the OM permeability barrier.

As the presence of ECA_CYC_ leads to damage to the OM permeability barrier in the absence of YhdP, we then asked whether, in wild-type cells, ECA_CYC_ plays a broader role in maintaining the OM permeability barrier. With high-throughput studies, the specificity of effects caused by insertions in the *wec* operon can be unclear, as these mutations, including those in *wzzE*, can be polar, causing loss of all ECA species (28). Thus, we investigated changes to envelope permeability with clean *wzzE* deletion.

**FIG 6:**
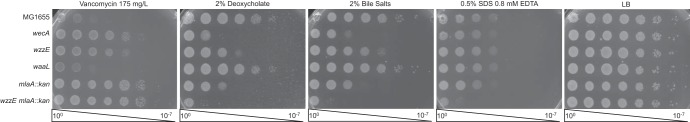
ECA_CYC_ plays a role in maintaining the OM permeability barrier. To assay changes to the OM due to removal of ECA_CYC_, EOPs were performed on strains with the indicated deletions. Removing ECA_CYC_ is responsible for some of the changes to the OM permeability barrier caused by ECA deletions. Combining *wzzE* and *mlaA* deletions caused synthetic SDS EDTA sensitivity. Images are representative of three independent experiments.

Cells with *ΔwzzE* showed a level of vancomycin resistance that was higher than that in *wzzE*^*+*^ cells and was, in fact, equal to that of *ΔwecA* cells (Fig. 6). The vancomycin resistance was similar to that observed with deletion of *mlaA*. MlaA is the first protein in the Mla pathway, which facilitates retrograde phospholipid transport and is responsible for preventing phospholipids from accumulating in the outer leaflet of the OM (59). In addition to vancomycin resistance, *ΔwzzE* cells also show sensitivity to deoxycholate, a detergent derived from bile salts, although less than that observed in *ΔwecA* cells. Deletion of *waaL*, which prevents formation of ECA_LPS_, had no effect on vancomycin resistance or DOC sensitivity. As WaaL does not influence deoxycholate resistance, these data suggest that both ECA_PG_ and ECA_CYC_ contribute to the deoxycholate phenotype, while ECA_CYC_ is responsible for vancomycin phenotype. Interestingly, combining strain *ΔwzzE* and *mlaA* deletion leads to an increase in SDS EDTA, deoxycholate, and bile salt sensitivity over that observed with either parent strain. This increase in detergent sensitivity suggests that combining these deletions causes larger changes to the OM than result from the individual mutations. Overall, these data demonstrate removal of ECA_CYC_ causes clear changes to the OM permeability barrier.

## DISCUSSION

In this work, we have established that ECA_CYC_ helps to maintain the OM permeability barrier and that YhdP controls this activity of ECA_CYC_ in such a way as to prevent damage to the OM. ECA_CYC_, not the membrane-associated form of ECA, is responsible for some of the OM permeability phenotypes caused by removal of ECA. Furthermore, in a *ΔyhdP* background, uncontrolled aberrant activity of ECA_CYC_ causes envelope permeability despite the fact that removing YhdP greatly lowered levels of ECA_CYC_. This role in maintenance of the OM permeability barrier is the first phenotype described for ECA_CYC_.

ECA is conserved throughout *Enterobacteriaceae* despite one of the OM forms acting as a surface-exposed common antigen that can lead to antibody production (29). Therefore, ECA must perform cellular functions that justify not only the risk of expressing a common antigen but also the potential for damage caused by ECA_CYC_. For the surface-exposed forms of ECA, one can imagine roles relating to direct host interactions, such as receptor binding interactions, interactions with other members of *Enterobacteriaceae*, or roles directly influencing the penetration of toxic substances into the cell; however, it is very difficult to imagine that ECA_CYC_, from its location in the periplasm, is responsible for interacting with the environment. Instead, ECA_CYC_ must play a role intrinsic to the cell. The changes in OM permeability that occur with removal of ECA_CYC_ demonstrate that ECA_CYC_ plays a role in maintaining the barrier function of the OM.

ECA_CYC_, as a cyclic soluble molecule made of aminosugars, has some resemblance to cyclodextrins. Cyclodextrins are cyclic carbohydrates made of glucose monomers that have a hydrophilic exterior and a hydrophobic cavity that allows them to bind to hydrophobic guests to increase their solubility and decrease their volubility (60). In fact, some cyclodextrins can pull specific molecules, such as cholesterol, out of membranes without binding to or disrupting the membranes (61–63). These properties have led to their use in drug formulations, as food additives, in cosmetics, as air deodorizers, and in many other applications (64). It is tempting to speculate that ECA_CYC_ may have similar properties allowing it to bind to specific target molecules in the periplasm and transfer them to or from the OM. In this case, it may be that YhdP is responsible for controlling what molecules are bound or where and how the molecules are unloaded.

Despite the large size of YhdP and strong phenotypes caused by its removal (36, 38), it is not apparent that YhdP has any unique role independent of ECA_CYC_, emphasizing the importance to the cell of controlling ECA_CYC_ activity. This also makes YhdP an important tool allowing for investigation of the effects of uncontrolled ECA_CYC_ in order to elucidate its normal function. The decrease in ECA_CYC_ in the *ΔyhdP* strain likely reflects a cellular mechanism to decrease the OM damage due to ECA_CYC_ in the absence of YhdP. The cell compensates for the loss of YhdP either through decreased synthesis or increased degradation of ECA_CYC_ to minimize OM damage. It is also possible that some ECA_CYC_ may leak out of *ΔyhdP* cells due to the OM damage; however, the methods for detecting ECA_CYC_ make this very difficult to determine. Nevertheless, even the low levels of ECA_CYC_ remaining are capable of damaging the OM barrier when its activity is uncontrolled. In the future, it will be of interest to investigate the mechanisms through which ECA levels are regulated. Nevertheless, our data suggest that, in the absence of ECA_CYC_ downregulation, the phenotypes of the *ΔyhdP* strain would be extremely severe.

Although the structure and topology of YhdP have not been experimentally determined, it is predicted to be an inner membrane protein with an N-terminal and possibly a C-terminal transmembrane helix, with the remainder of the protein exposed in the periplasm (37). YhdP is classified as a member of the AsmA family of proteins due to the presence of a C-terminal AsmA_2 domain and an N-terminal DUF3971 domain, which contain shared sequence motifs with those found in AsmA (58). In E. coli, there are six members of this family, AsmA, YhdP, TamB (YtfN), YhjG, YicH, and YdbH (65). Although the function of these proteins is largely unknown, mutations in *asmA* have been found to suppress assembly-defective mutations in OMPs (66, 67). In addition, TamB has been suggested to interact with the OMP TamA to allow secretion of autotransporters (68). Half of the DUF490 domain of TamA has been crystallized and found to adopt a “taco-shaped” β-sheet with a hydrophobic cavity (69). The remainder of the protein is thought to adopt a similar conformation, perhaps allowing amphipathic OMP segments to be transferred to TamA to avoid the aqueous periplasm. This structure is similar to that of the β-jellyroll conformation found in the LptA protein responsible for LPS transport across the periplasm (69, 70).

Although TamA and YhdP share only 25% identity, it is possible that YhdP adopts a similar conformation allowing it to bind to hydrophobic molecules. If this is the case, then YhdP may bind to hydrophobic molecules and pass them to ECA_CYC_ or unload molecules from ECA_CYC_. We are currently investigating the specific mechanisms and pathways through which YhdP and ECA_CYC_ act, including the possibility that YhdP and ECA_CYC_ may interact physically. Nevertheless, the functional interaction between YhdP and ECA_CYC_ and the strong phenotype caused by ECA_CYC_ in the absence of YhdP represent an important aspect to envelope biology that has yet to be explored.

The difference in cellular location between ECA_CYC_ and the other forms of ECA, the ability of the cell to make three forms of ECA, and the differing antibiotic sensitivities with removal between these forms, suggests that the function of ECA_CYC_ and YhdP are likely not the same as the functions of ECA_PG_ and ECA_LPS_. In addition, while ECA_PG_ has a role, direct or indirect, in excluding toxic substances, ECA_LPS_ appears to have no role in maintaining the OM permeability barrier and may instead have a role in interacting with the environment or be a by-product of the reaction that attaches O antigen to LPS. Interestingly, when investigating changes to the various forms of ECA in the presence and absence of *yhdP*, we observed that the modal chain length of the membrane-bound forms of ECA varied based on temperature. Knowledge on changes in ECA chain length in response to temperature has not been reported. This change in chain length may be due to specific regulation of ECA length by temperature or by a temperature-dependent change in the activity of the ECA polymerase, WzyE. However, in Yersinia enterocolitica, expression of ECA has been found to be modulated by temperature changes, with high levels of ECA at 22°C and almost undetectable levels at 37°C (71, 72). These data suggest that the functional requirements for the membrane-bound forms of ECA may depend on temperature. Furthermore, the differences in regulation of ECA expression between genera in *Enterobacteriaceae* suggest that the role of ECA may be adapted or modified for the lifestyles of different species. Investigation of these differences may lead to interesting insights into the biology of these species.

## MATERIALS AND METHODS

### Strains and growth conditions.

The strains used in this work are listed in Table S2. Cultures were grown at 37°C in LB medium unless otherwise noted. When necessary, cultures were supplemented with 20 mg/liter chloramphenicol, 25 mg/liter kanamycin, or 25 mg/liter tetracycline. To quantitate ECA_CYC_ levels, cells were grown in M63 medium without nitrogen and supplemented with 0.2% glucose, 0.2% (NH_4_)_2_SO_4_, 1 mM MgSO_4_, and 100 µg/ml thiamine. Deletion alleles originated from the Keio collection (73), unless otherwise noted, and were moved into our strains by P1*vir* transduction (74). Unless otherwise indicated, resistance cassettes were flipped out as has been described previously (75).

10.1128/mBio.01321-18.10 TABLE S2  Strains used in this study. Download TABLE S2, DOCX file, 0.03 MB. Copyright © 2018 Mitchell et al. 2018 Mitchell et al. This content is distributed under the terms of the Creative Commons Attribution 4.0 International license.

### Antibiotic sensitivity assays.

For growth curves, overnight cultures were diluted 1:1,000 into 2 ml fresh LB containing the compounds indicated in a 24-well format, sealed with breathable film, and grown shaking at 37°C in a BioTek Synergy H1 plate reader. The optical density at 600 nm (OD_600_) was assayed every 10 min. MICs were determined as has been reported elsewhere (57). The MIC was taken to be the minimum concentration of antibiotic at which no growth was observed. For efficiency of plating (EOP) assay, 10-fold dilutions of overnight cultures were made and replicate plated onto LB plates supplemented with the indicated chemicals. Plates were incubated at 30°C (unless otherwise noted) overnight and plates were imaged.

### Generation and mapping of suppressor mutations.

To generate spontaneous suppressor mutants, we plated 10^7^ *ΔyhdP* strain cells on LB supplemented with 70 mg/liter vancomycin and incubated the plates overnight at 30°C. Colonies were picked and subjected to secondary screening for vancomycin and SDS EDTA resistance. The suppressor mutations were mapped as has been described elsewhere (76). We generated a Tn*5* mutant library in a *ΔyhdP* strain as has been described elsewhere (36). Our selection and screening strategy for isolating suppressing mutations is outlined in Fig. S3A. The transposon insertion site were determined by arbitrary PCR as has been described previously (59), except that the TetA-out and TetA-seq primers were replaced with Tn5-out (5′ GGTTGTAACACTGGCAGAGC 3′) and Tn5-seq (5′ TCCGTGGCAAAGCAAAAGTT 3′).

### Phylogenetic co-occurrence and homologies.

To determine whether *yhdP* and the genes of the *wec* operon tend to occur in the same genomes across organisms, we utilized the co-occurrence channel of STRING-DB (39, 77). We searched the database in multiple-protein mode for *yhdP* and ECA biosynthesis genes and took the phylogenetic co-occurrence scores from the generated table. The derivation of these scores from homology tables has been described elsewhere (77). To examine the level of homology for possible YhdP homologues, we used the homology scores generated via the STRING database to find whether the indicated classification of organisms was predicted to have an YhdP homology and what the highest and lowest homology scores were for the organisms included in STRING-DB within that classification. These scores were then plotted.

### σ^E^ reporter assay.

To determine the level of activation of the σ^E^ system, we utilized a plasmid reporter with the promoter from *micA* driving expression of GFP (57) as has been reported elsewhere (78). Each of the three independent experiments was conducted in technical triplicate. The significance of the differences observed was calculated using the nonparametric Mann-Whitney test.

### Quantification of ECA levels.

Membrane-associated forms of ECA were analyzed by immunoblot analysis. Cells from an overnight culture were resuspended in BugBuster protein extraction reagent (Millipore Sigma) at an equivalent OD_600_ of 40 and then combined with an equal volume of Laemmli sample buffer (Bio-Rad) with 4% β-mercaptoethanol. Samples were boiled 5 min and then cooled and loaded on 12% TGX gels (Bio-Rad). The samples were transferred to nitrocellulose and were probed with a 1:10,000 dilution of anti-ECA antibody. Rabbit polyclonal anti-ECA antibody was a kind gift from Renato Morona (University of Adelaide). Donkey anti-rabbit secondary antibody conjugated to horseradish peroxidase was utilized at a 1:20,000 dilution and detected using a Crescendo ECL system (Millipore Sigma). The specificity of the ECA antibody could be observed based on the lack of signal with the *ΔwecA* strain (Fig. 4B, lanes 1 and 6). Levels of ECA were quantitated using ImageJ. Densitometry was performed on blots with the lowest exposure at which the ECA bands for the indicated samples could be detected. Densitometry was performed on the whole lane and manually baselined. Similar results were found when each ECA band was measured individually. For each of three to five biological replicates, fold values to the *yhdP*^*+*^ sample were calculated. Then, the biological replicates were averaged and the standard errors of the means (SEM) were calculated. Significance was calculated using the Mann-Whitney test.

ECA_CYC_ was purified as has been described before, with minor modifications (34). Cells were grown in LB medium for determination of the ECA_CYC_ structure. For determination of ECA_CYC_ levels, cells were grown in M63 medium with either a normal or heavy (^15^N) nitrogen source, and cultures for comparison were combined at the beginning of purification. After ethanol precipitation, supernatants were lyophilized and subsequently resuspended with 0.1% formic acid. Acidified samples were loaded on C_18_ StageTips (79), washed twice with 0.1% formic acid, and eluted with 20% acetonitrile with 0.1% formic acid. Eluates were then dried in a Speedvac before reconstitution with 20% acetonitrile. Samples were analyzed by MALDI-TOF/mass spectroscopy as has been previously described (34). Spectra were obtained with a Bruker UltrafleXtreme instrument calibrated with Red phosphorous. For relative quantification, the ratio of the areas of the heavy and normal ECA_CYC_ peaks was calculated for three biological replicates. Significance was calculated using the Mann-Whitney test.
