# Supplementary material for: Cyclic Enterobacterial Common Antigen Maintains the Outer Membrane Permeability Barrier of Escherichia coli in a Manner Controlled by YhdP
Source: mBio. 2018 Aug 7;9(4):e01321-18. doi: 10.1128/mBio.01321-18 (PMC6083912; doi:10.1128/mBio.01321-18)
Supplement: TABLE S1 [file mbo004184012st1.docx]

**Table S1: Phenotypes of insertions isolated from transposon screen**

| Group | Gene | Total  clones | Unique insertions | Vanc Resistance | SDS EDTA Resistance |
| --- | --- | --- | --- | --- | --- |
| Control strains | **MG1655** | **--** | **--** | **++++** | **++++++** |
|  | **Δ*yhdP*** | **--** | **--** | **-** | **+++** |
| Δ*yhdP*  background | *wecA* | 11 | 8 | ++++++ | +++++ |
|  | *wzzE* | 8 | 6 | ++++++ | +++++ |
|  | *wecB* | 3 | 3 | ++++++ | +++++ |
|  | *wecC* | 5 | 4 | ++++++ | +++++ |
|  | *rffH* | 1 | 1 | ++++++ | +++++ |
|  | *wecD* | 1 | 1 | ++++++ | +++++ |
|  | *wecE* | 6 | 5 | ++++++ | +++++ |
|  | *wecF* | 8 | 2 | ++++++ | +++++ |
|  | *wecG* | 4 | 2 | ++++++ | +++++ |
|  | *dusB* | 1 | 1 | + | +++ |
|  | *pdeK* promoter | 1 | 1 | ++++++ | ++ |
|  | *opgG* | 1 | 1 | ++++ | ++ |
|  | *opgH* | 2 | 2 | ++++ | ++ |
|  | *fimD* | 1 | 1 | ++ | +++ |
|  | *php* | 7 | 1 | ++++++ | +++ |
|  | *phoP* | 1 | 1 | + | **-** |
|  | *phoQ* | 1 | 1 | + | **-** |
|  | *mlaA* | 1 | 1 | ++ | **-** |
|  | *mlaE* | 1 | 1 | ++ | **-** |
|  | *tolC* | 8 | 6 | +++ | **-** |
|  | *qseC* | 1 | 1 | +++ | **-** |
|  | *dacA* | 1 | 1 | +++ | ++ |
|  | *mltG* | 1 | 1 | ++ | +++ |
|  | *prc* | 2 | 2 | +++ | + |
|  | *nlpI* | 1 | 1 | ++ | + |
|  | *yiiX* | 1 | 1 | + | +++ |
|  | *mutS* | 4 | 4 | +++ | +++ |
|  | *mutL* | 7 | 6 | +++ | +++ |
|  | *radA* | 2 | 1 | ++++ | +++ |
|  | *yicC* | 1 | 1 | + | +++ |
|  | *uvrD* | 1 | 1 | + | +++ |
