# Supplementary figures and images for: Cyclic Enterobacterial Common Antigen Maintains the Outer Membrane Permeability Barrier of Escherichia coli in a Manner Controlled by YhdP
Source: mBio. 2018 Aug 7;9(4):e01321-18. doi: 10.1128/mBio.01321-18 (PMC6083912; doi:10.1128/mBio.01321-18)

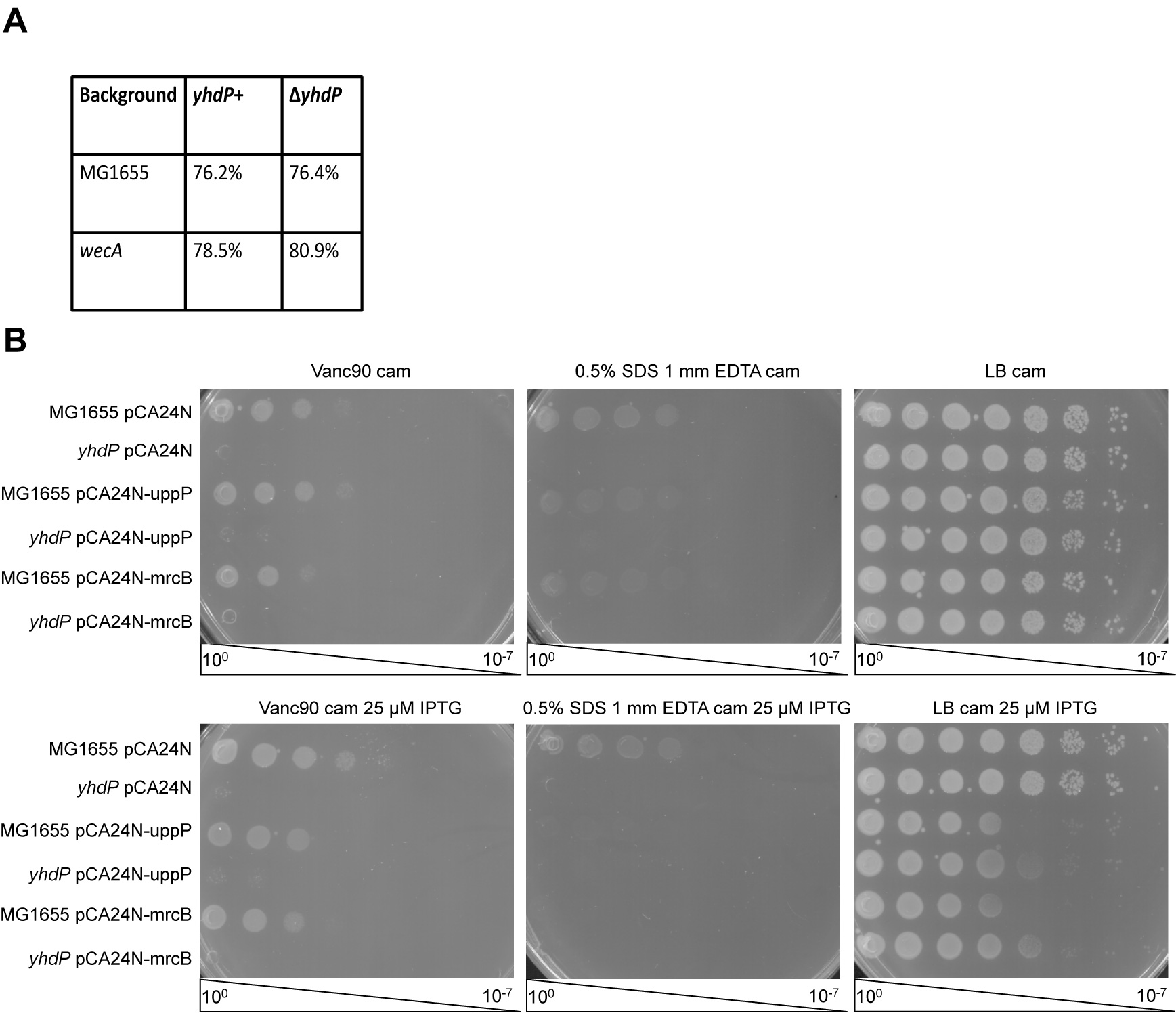

Supplement: FIG S4 [file mbo004184012sf4.jpg]

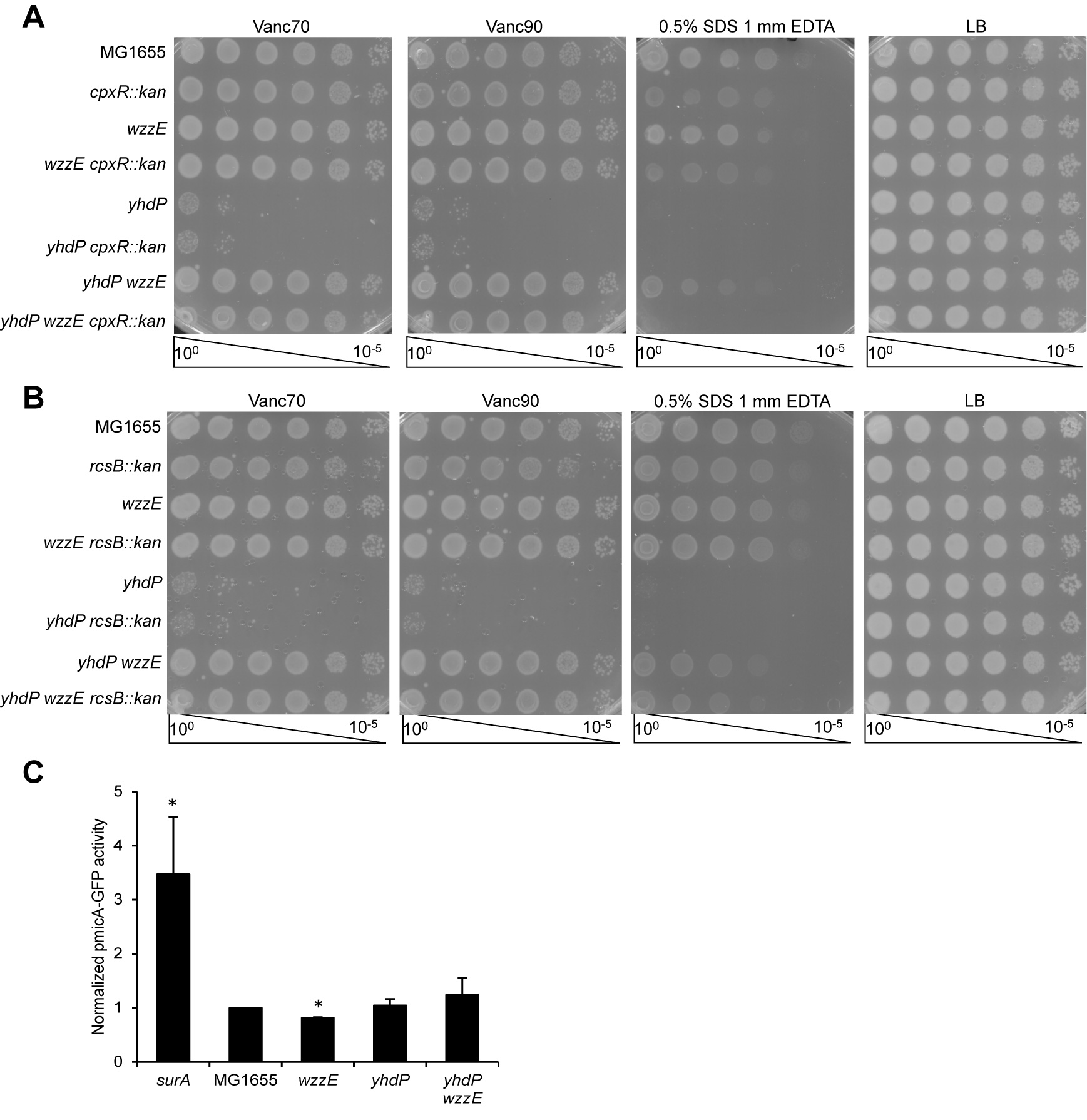

Supplement: FIG S8 [file mbo004184012sf8.jpg]
