# Supplementary material for: Cyclic Enterobacterial Common Antigen Maintains the Outer Membrane Permeability Barrier of Escherichia coli in a Manner Controlled by YhdP
Source: mBio. 2018 Aug 7;9(4):e01321-18. doi: 10.1128/mBio.01321-18 (PMC6083912; doi:10.1128/mBio.01321-18)
Supplement: TABLE S2 [file mbo004184012st2.docx]

**Table S2: Strains used in this study**

| Strain | Genotype | Reference |
| --- | --- | --- |
| MG1655 | K-12 F^-^ λ^-^ *rph-1* | (1) |
| AM182 | MG1655 *ΔyhdP* | (2) |
| AM328 | MG1655 *ΔyhdP* WecA^A57ins[IS2]^ (nucleotide 172 ins[IS2]) | This study |
| AM329 | MG1655 *ΔyhdP* WecA^G58ins[IS1]^ (nucleotide 174 ins[IS1]) | This study |
| AM330 | MG1655 *ΔyhdP* WecA^F93LfsX5^ (del nucleotide 276) | This study |
| AM331 | MG1655 *ΔyhdP* WecC^S125X^ (nucleotide 373-375 TCA>TAA) | This study |
| AM332 | MG1655 *ΔyhdP* WecC^P238-G239dup^ (nucleotide 778 ins[CTGGCC]) | This study |
| AM333 | MG1655 *ΔyhdP* WecF^I336ins[IS30]^ (nucleotide 1009 ins[IS30]) | This study |
| AM174 | MG1655 *wecA::kan* | (2) |
| Am306 | MG1655 *bamE::cam* | This study  *bamE* allele (3) |
| AM307 | MG1655 *bamE::cam wecA::kan* | This study  *bamE* allele (3) |
| AM304 | MG1655 *ΔyhdP wecA::kan* | This study |
| AM362 | MG1655 *ΔyhdP ΔwaaL ilvD::Tn10* | This study  *ilvD* allele (4) |
| AM370 | MG1655 *ΔyhdP ΔwaaL* | This study |
| AM334 | MG1655 *ΔwecA* | This study |
| AM337 | MG1655 *ΔwecE* | This study |
| AM341 | MG1655 *ΔyhdP ΔwecA* | This study |
| AM344 | MG1655 *ΔyhdP ΔwecE* | This study |
| AM046 | MG1655 *cpxR::kan* | This study |
| AM336 | MG1655 *ΔwecA cpxR::kan* | This study |
| AM340 | MG1655 *ΔyhdP cpxR::kan* | This study |
| AM343 | MG1655 *ΔyhdP ΔwecA cpxR::kan* | This study |
| AM049 | MG1655 *rcsB::kan* | This study |
| AM335 | MG1655 *ΔwecA rcsB::kan* | This study |
| AM339 | MG1655 *ΔyhdP rcsB::kan* | This study |
| AM342 | MG1655 *ΔyhdP ΔwecA rcsB::kan* | This study |
| AM346 | MG1655 pAYC184micA-GFP | This study  Plasmid (5) |
| AM347 | MG1655 *surA::kan* pACYCmicA-GFP | This study  Plasmid (5) |
| AM348 | MG1655 *ΔwecA* pACYCmicA-GFP | This study  Plasmid (5) |
| AM349 | MG1655 *ΔwecA* pACYCmicA-GFP | This study  Plasmid (5) |
| AM351 | MG1655 *ΔyhdP* pACYCmicA-GFP | This study  Plasmid (5) |
| AM352 | MG1655 *ΔyhdP ΔwecA* pACYCmicA-GFP | This study  Plasmid (5) |
| AM353 | MG1655 *ΔyhdP ΔwecE* pACYCmicA-GFP | This study  Plasmid (5) |
| AM365 | MG1655 *ΔwzzE* | This study |
| AM366 | MG1655 *ΔwaaL* | This study |
| AM395 | MG1655 *ΔwzzE ΔwaaL* | This study |
| AM369 | MG1655 *ΔyhdP ΔwzzE* | This study |
| AM396 | MG1655 *ΔyhdP ΔwzzE ΔwaaL* | This study |
| AM397 | MG1655 *wecH::kan* | This study |
| AM398 | MG1655 *ΔyhdP wecH::kan* | This study |
| Am179 | MG1655 *wecE::kan* | (2) |
| AM305 | MG1655 *ΔyhdP wecE::kan* | This study |
| AM476 | MG1655 pCA24N | This study  Plasmid (6) |
| AM477 | MG1655 *ΔyhdP* pCA24N | This study  Plasmid (6) |
| AM478 | MG1655 pCA24N-uppS | This study  Plasmid (6) |
| AM479 | MG1655 *ΔyhdP* pCA24N-uppS | This study  Plasmid (6) |
| AM482 | MG1655 pCA24N-murA | This study  Plasmid (6) |
| AM483 | MG1655 *ΔyhdP* pCA24N-murA | This study  Plasmid (6) |
| AM480 | MG1655 pCA24N-uppP | This study  Plasmid (6) |
| AM481 | MG1655 *ΔyhdP* pCA24N-uppP | This study  Plasmid (6) |
| AM484 | MG1655 pCA24N-mcrB | This study  Plasmid (6) |
| AM485 | MG1655 *ΔyhdP* pCA24N-mcrB | This study  Plasmid (6) |
| MG1635 | F^−^ *araD139* λ^−^ *rph-1 zad-220::Tn10 mrcB::kan* | (7) |
| AM368 | MG1655 *ΔwzzE cpxR::kan* | This study |
| AM372 | MG1655 *ΔyhdP ΔwzzE cpxR::kan* | This study |
| AM367 | MG1655 *ΔwzzE rcsB::kan* | This study |
| AM371 | MG1655 *ΔyhdP ΔwzzE rcsB::kan* | This study |
| AM350 | MG1655 *ΔwzzE* pACYCmicA-GFP | This study  Plasmid (5) |
| AM354 | MG1655 *ΔyhdP ΔwzzE* pACYCmicA-GFP | This study  Plasmid (5) |
| AM522 | MG1655 *mlaA::kan* | This study |
| AM523 | MG1655 *ΔwzzE mlaA::kan* | This study |

**References**

1. Guyer MS, Reed RR, Steitz JA, Low KB. 1981. Identification of a sex-factor-affinity site in E. coli as gamma delta. Cold Spring Harb Symp Quant Biol 45 Pt 1:135-40.

2. Mitchell AM, Wang W, Silhavy TJ. 2017. Novel RpoS-Dependent Mechanisms Strengthen the Envelope Permeability Barrier during Stationary Phase. J Bacteriol 199.

3. Ricci DP, Hagan CL, Kahne D, Silhavy TJ. 2012. Activation of the Escherichia coli beta-barrel assembly machine (Bam) is required for essential components to interact properly with substrate. Proc Natl Acad Sci U S A 109:3487-91.

4. Singer M, Baker TA, Schnitzler G, Deischel SM, Goel M, Dove W, Jaacks KJ, Grossman AD, Erickson JW, Gross CA. 1989. A collection of strains containing genetically linked alternating antibiotic resistance elements for genetic mapping of Escherichia coli. Microbiol Rev 53:1-24.

5. Konovalova A, Grabowicz M, Balibar CJ, Malinverni JC, Painter RE, Riley D, Mann PA, Wang H, Garlisi CG, Sherborne B, Rigel NW, Ricci DP, Black TA, Roemer T, Silhavy TJ, Walker SS. 2018. Inhibitor of intramembrane protease RseP blocks the sigma(E) response causing lethal accumulation of unfolded outer membrane proteins. Proc Natl Acad Sci U S A doi:10.1073/pnas.1806107115.

6. Kitagawa M, Ara T, Arifuzzaman M, Ioka-Nakamichi T, Inamoto E, Toyonaga H, Mori H. 2005. Complete set of ORF clones of Escherichia coli ASKA library (a complete set of E. coli K-12 ORF archive): unique resources for biological research. DNA Res 12:291-9.

7. Grabowicz M, Andres D, Lebar MD, Malojcic G, Kahne D, Silhavy TJ. 2014. A mutant *Escherichia coli* that attaches peptidoglycan to lipopolysaccharide and displays cell wall on its surface. Elife 4:e05334.
